# Supplementary material for: Identification of Circular RNAs in the Anterior Pituitary in Rats Treated with GnRH
Source: Animals (Basel). 2021 Aug 31;11(9):2557. doi: 10.3390/ani11092557 (PMC8466137; doi:10.3390/ani11092557)
Supplement: Supplementary file 1 [file animals-11-02557-s001.zip › Support file 1.pdf]

COMMITTEE FOR VETERINARY MEDICINAL PRODUCTS

GONADOTROPHIN RELEASING HORMONE (GONADORELIN)

SUMMARY REPORT

1. Gonadorelin is a synthetic decapeptide with a structure identical with the natural Gonadotrophin Releasing Hormone (Gn-RH) in mammalian species.

2. Gonadorelin is recommended for the treatment of reproductive disorders associated with ovarian cysts and for improvement of fertility in cows and for the induction of ovulation in rabbits. The mechanism of action underlying the therapeutic use is the stimulation of the release from and also the synthesis of LH and FSH in the anterior pituitary gland.

3. The pituitary LH and FSH release in response to exogenous Gn-RH was investigated in guinea pigs, rats, rabbits, monkeys, sheep, mares and cows. In all animal species studied, parenteral administration of Gn-RH resulted in a distinct increase in plasma LH and FSH levels. From a rat study (s.c.) using gonadorelin, an ED50 of about 80 ng/animal was derived. The full effective dose was estimated to be 150-200 ng/animal, a value in accordance with literature data.

4. In cattle, the increase in plasma LH and FSH was demonstrated to occur in all stages of the oestrous cycle. LH release was dose dependent and varied with the stage of cycle reflecting the inhibitory/stimulating influences of steroid hormones. The administration of the recommended i.m. dose range of 0.1-0.5 mg gonadorelin/cow causes an immediate and substantiated increase in plasma LH and FSH levels. The LH peaks are within the range of physiological preovulatory peak levels.

   In rats, intragastric or sublingual administration of gonadorelin was effecive in inducing ovulation at exceedingly high experimental doses ranging from 500-1000 µg/rat or 50-500 µg/rat respectively. For both modes of application a No Observed Effect Level could not be established. Referring to a previous rat study, from which a fully effective s.c. dose of 0.15-0.2 µg/animal was derived, the activity ratio of gonadorelin following subcutaneous, sublingual and intragastric adiministration is estimated approximately 1:250:2500. Thus it is suggested that the oral activity of gonadorelin is negligible when compared to the subcutaneous route.

5. The pharmacokinetics of radio labelled Gn-RH were investigated in rats (i.v.), mice (i.v.), rabbits (i.v.) and cattle (i.v., i.m.). In all species studied, the biological half-life of gonadorelin was found to be about 4 minutes follwing i.v. administration. After i.m. administration in cows, gonadorelin was rapidly absorbed from the injection site, with a plasma half-life of approximately 20 minutes. Distribution studies in rats revealed greater Gn-RH concentrations in the pineal gland, anterior and posterior pituitaries, ovaries, liver and kidney than in plasma. The compound is rapidly metabolised into smaller inactive peptides and animoacids. The major pathways of excretion are urine and expired air.

6. A single dose toxicity study performed in mice (i.v.) revealed no toxic effects of Gn-RH at doses ranging from 1-50 mg/kg bw.

7 Westferry Circus, Canary Wharf, London E14 4HB, UK
Tel: (+44-171) 418 84 00 Fax: (+44-171) 418 84 16

7. No data on single or repeated dose toxicity, reproductive toxicity and teratogenicity, mutagenicity and carcinogenicity were provided. However with respect to the properties of the compound, as mentioned below, the submitted data is regarded as sufficient for a conclusive assessment of this compound :

   * from the i.m. injection site Gn-RH is readily absorbed, rapidly metabolised and excreted; no residues were found at the injection site 24 hours post-injection;

   * following oral administration, gonadorelin is rapidly degraded in the gastrointestinal tract. As concluded from the a.m. rat study, traces of the active compound can be absorbed through the mucosa of the mouth or gut following application of high doses. However, since residue levels in food derived from clinically treated animals are very low, gonadorelin is considered not to pose any risk on the consumer.

8. It is proposed to insert gonadorelin into Annex II of Council Regulation (EEC) n° 2377/90 for all food producing species.
